# Association of *Helicobacter pylori* Infection with Autoimmune Thyroid Disease in the Female Sex

**DOI:** 10.3390/jcm12155150

**Published:** 2023-08-06

**Authors:** Maria Pina Dore, Giuseppe Fanciulli, Alessandra Manca, Giovanni Mario Pes

**Affiliations:** 1 Dipartimento di Medicina, Chirurgia e Farmacia, University of Sassari, 07100 Sassari, Italyalessandra.manca@aouss.it (A.M.);; 2 Baylor College of Medicine, Houston, TX 77030, USA; 3 Sardinia Blue Zone Longevity Observatory, 08040 Santa Maria Navarrese, Italy

**Keywords:** autoimmune thyroid disease, Hashimoto’s thyroiditis, Graves’ disease, *Helicobacter pylori*

## Abstract

Background. *Helicobacter pylori* infection has been associated with an increased risk of thyroid diseases (TDs), although scientific evidence is conflicting. In the present study the relationship between TDs, including both autoimmune (AI) and non-autoimmune TD, and *H. pylori* infection was investigated. Methods: Data from records of patients undergoing upper endoscopy and histologically evaluated for *H. pylori* infection were retrieved. In addition to demographic information, the features of gastritis based on non-targeted biopsies collected from the antrum, angulus, and corpus were analyzed. The presence of *H. pylori* infection and atrophy and/or metaplasia and/or dysplasia in at least one gastric specimen was defined as a long-lasting *H. pylori* infection and the presence of a chronic–active gastritis as a current infection. Hashimoto’s and Graves’ diseases were included in the AITD group, and thyroid nodules, goiter, iatrogenic thyroid hypo/hyper function, and thyroidectomy in the non-autoimmune TD group. Results: A total of 8322 records from adult patients from Northern Sardinia, characterized by a similar genetic background, was analyzed. Participants were aged 18–93 years (females 5339, 64.1%), and more specifically, 562 (6.7%) had a diagnosis of AITD and 448 (5.4%) of non-autoimmune TD. A significant association between long-lasting *H. pylori* and AITD (OR 1.34; 95%CI 1.13–1.60) was found, irrespective of age, sex, body mass index, and smoking status, while it was not associated with non-autoimmune TD. Current *H. pylori* infection did not show significant ORs for AITD (OR 0.99; 95%CI 0.64–1.57) and non-autoimmune TD (OR 0.86; 95%CI 0.66–1.15). The association with long-lasting *H. pylori* infection was confirmed to be significant for both Hashimoto’s thyroiditis and Graves’ disease by multivariable regression analysis. Stratification according to sex revealed a significant association only for females (OR 1.39; 95%CI 1.12–1.72). Conclusions. Our results indicate that long-lasting *H. pylori* infection is associated with AITD in the female adult population of Northern Sardinia.

## 1. Introduction

*Helicobacter pylori* (*H. pylori*) is a flagellated Gram-negative bacterium mainly transmitted via the fecal–oral route and colonizing approximately half of the world’s population [1]. The pathogen is adapted to the gastric environment, which represents the main target of the infection. Unlike other Gram-negative bacteria, which elicit an acute reaction in the host capable of containing the infection and clearing the bacterium, the 60,000 year evolution-driven adaptation strategy of *H. pylori* to the host’s environment [2] relies on the attenuation of the host’s innate and adaptive immune response [3]; therefore, most infection carriers are asymptomatic or paucisymptomatic [4,5] and present with dyspepsia-like complaints [6]. Although most *H. pylori* infections are asymptomatic, the infection is not benign. The bacterial infection of the gastric mucosal surface causes progressive damage to the gastric mucosa with eventual impairment of gastric function, and in the long run, peptic ulcer, gastric cancer, and mucosa-associated lymphoid tissue (MALT) lymphoma may develop [7]. *H. pylori* infection is typically acquired in childhood; the high proportion of older individuals who are infected is the result of infection during childhood, when standards of living were lower (a birth cohort phenomenon) [8]. The bacteria selectively attaches to the surface of mucus-secreting cells of the stomach through complex adhesin molecules [9], being the site of adherence to the intercellular junctions of the epithelial cells. If not properly diagnosed and treated [10,11,12], the natural history of the *H. pylori* infection is characterized by the appearance of atrophy, metaplasia, and dysplasia based on specific characteristics of the bacterium, the host, and the environment. While *H. pylori* is not considered an invasive pathogen, it can be found within epithelial cells, suggesting that an intracellular location may serve as a niche site for evasion of the host immune defenses [13]. During the past few decades, research on *H. pylori* has indicated that despite being confined to the stomach, the infection may also cause extraintestinal manifestations. For instance, *H. pylori* eradication is recommended in cases of unexplained iron deficiency anemia, idiopathic thrombocytopenic purpura, and deficiency of vitamin B12 [6]. Several investigations have tried to identify key pathogenetic mechanisms suggesting the microorganism as a systemic pathogen [14]. Specifically, the remodeling of the host’s immune response, enabling *H. pylori* to escape early clearance and promoting its survival in the host environment, may enhance, as a side effect, the risk of developing organ-specific autoimmune manifestations such as Behçet disease [15], type-1 diabetes mellitus [16], rheumatic disorders [17], and systemic lupus erythematosus [18], to mention a few [19]. In particular, *H. pylori* has been implicated in autoimmune thyroid disorders (AITDs) [20] as well as in a number of non-autoimmune TDs. This seems to have been confirmed by reduced anti-thyroid antibody titers following eradication therapy for *H. pylori* [21]. In a group of patients with an anti-thyroid peroxidase (TPO titer > 700 IU/mL) (normal value 0–35 IU/mL) and *H. pylori* infection confirmed by the 13C urea breath test (UBT), titers of anti-TPO and anti-thyroglobulin antibodies, after successful eradication and follow-up for 17–24 months, all showed remarkable reduction in anti-TPO titers (*p* < 0.001, χ2 test).

In fact, an inflammatory component is associated both with *H. pylori* [22] infection and with thyroid diseases [23], including non-autoimmune thyroid disease, representing the pathogenetic basis for an association between the two clinical conditions. In the past twenty years, the potential of *H. pylori* infection to increase the risk of AITD was explored in several surveys, obtaining mixed results. The majority of cross-sectional studies support the association between *H. pylori* infection and increased AITD risk across Western [24,25,26,27,28], Middle-Eastern [29], and Asian populations [30]. More importantly, prospective studies investigating AITD over time in carriers of *H. pylori* infection [31], as well as two meta-analyses [32,33], have furnished evidence for a positive association. 

Hou et al., in a total of 3046 cases (1716 observational and 1330 control cases), found a positive and significant correlation between *H. pylori* infection and AITD (OR = 2.25, 95%CI: 1.72–2.93). The association was confirmed also for infection caused by cytotoxic bacteria (positive for cytotoxin-associated gene A (CagA)), albeit to a lesser extent (OR = 1.99, 95%CI: 1.07–3.70) [32].

Similarly, the meta-analysis conducted by Shi et al., in a total of 862 participants (466 cases and 396 controls), highlighted a significant association of *H. pylori* infection with AITDs (OR 1.92, 95%CI 1.41–2.61) [33]. However, in this study, the effect size of CagA seropositivity was significantly stronger for AITDs (OR = 2.24; 95%CI 1.06–4.75) [33]. Accordingly, virulent strains, identified by the presence of CagA, have been implicated in both organ-specific and non-organ-specific autoimmune diseases [17]. Other authors did not find any significant association [34]. Several methodological issues could explain these discrepancies, including the cohort’s small size [20], the diagnostic test used, the genetic background of the population investigated, and the lack of adjustment for potential confounding factors. Notably, the majority of studies considered only *H. pylori* infection in general without separating current infection (characterized by chronic–active inflammation) and long-standing infection (characterized by the presence of metaplasia and/or dysplasia and/or atrophy), although the pathogenetic underpinnings are quite different in the two conditions. Taking these premises into account, in the present study the association between TDs, both AITD and non-autoimmune TD, and *H. pylori* infection was explored in a cohort of subjects undergoing endoscopic examinations of the upper tract, and in whom the presence or absence of an *H. pylori* infection was ascertained, as were the histopathological features of gastritis.

## 2. Materials and Methods

### 2.1. Study Design and Data Collection

This was a retrospective case-control single-center study. Data were collected from clinical records of adult patients undergoing upper endoscopy for dyspeptic symptoms at the Gastroenterology Unit of the teaching hospital Clinica Medica of the University of Sassari, Italy. All participants were from Northern Sardinia, a white Caucasian population characterized by a homogenous genetic background [35] and a reported high frequency of autoimmune diseases and *H. pylori* infection [36]. For patients undergoing multiple endoscopies, only the index case was considered for the analysis. Information retrieved included demographic and anthropometric parameters, a medical history comprehensive of all defined diagnoses, and ongoing treatments. 

Due to the observational design of the study according to Italian law (GU No. 76 31/Mar/2008), ethics review and approval were waived.

### 2.2. Helicobacter Pylori Status

In each patient, at least four non-targeted biopsies were collected, if not contraindicated during endoscopy, more specifically, two from the antrum, one from the angulus, and one from the corpus. Briefly, gastric specimens were evaluated for morphology by a dedicated GI pathologist. The presence of *H. pylori* associated with areas of atrophy and/or metaplasia and/or dysplasia in at least one gastric sample was defined as a long-lasting *H. pylori* infection. Detection of chronic–active gastritis characterized by the presence of granulocytes and lymphocytes, in addition to the bacteria on specimens, was defined as a current infection. 

### 2.3. Diagnosis of Thyroid Disorders

The diagnosis of the TD was made by the endocrinologist according to national and international guidelines/expert consensus used in the clinical setting based on clinical features, positivity for serum antibodies against thyroid peroxidase (anti-TPO) and thyroglobulin, thyroid ultrasound, and, in specific cases, thyroid cytology as previously described [37]. TDs were classified into AITDs (Hashimoto thyroiditis and Graves’ disease) and non-autoimmune TDs (goiter, nodules, cancer, post-surgical hypothyroidism, and others) Moreover, TDs were classified according to gland functionality, categorized as hypothyroidism, hyperthyroidism, and euthyroidism.

### 2.4. Statistical Analysis

The SPSS statistical package was used to conduct the statistical analyses (SPSS 22, Chicago, IL, USA). Patients with TDs (cases) were compared to patients without TDs (controls) according to *H. pylori* status. Multivariable logistic regression analysis was used to estimate the risk of AITD and non-autoimmune TD, and long-lasting or current *H. pylori* infection, adjusting for confounding factors (i.e., sex, age, body mass index (BMI), and smoking status). Patients with celiac disease, a well-known increased risk factor for developing AITD [37], were excluded from the analysis. A two-tailed *p*-value < 0.05 was considered as the threshold for statistical significance.

## 3. Results

A total of 8322 records from patients aged 18–93 years was examined. Table 1 illustrates the overall characteristics of the cohort investigated. Briefly, the mean age of the cohort was 52.33 ± 16.91 years, and the percentage of females was greater than that of males (60.9% and 39.1%, respectively). Among 1010 (12.1%) patients with a diagnosis of TD, 562 (55.6%) patients had more specifically an AITD.

As expected, the proportion of individuals with TD showed a slightly increasing trend with age. Although mildly, excess weight was observed among patients with TD, while the percentage of current or former smokers did not differ between the three groups. *H. pylori* infection was confirmed in 3395 patients among a total of over 30,000 gastric specimens analyzed.

More specifically, a diagnosis of long-lasting infection was made in 1763 (51.9%) patients, and a diagnosis of current *H. pylori* infection in 1632 (48.1%) patients. A past *H. pylori* infection—successfully eradicated—was present in the clinical history of 316 patients. The proportion of subjects with long-lasting infection was significantly greater in the subgroup of patients with AITD (26.5% vs. 20.8%, *p* < 0.001), whereas no statistical difference was found regarding current (16.5% vs. 19.9%, n.s.) and past (4.8% vs. 3.5%, n.s.) infections.

**Figure 1:**
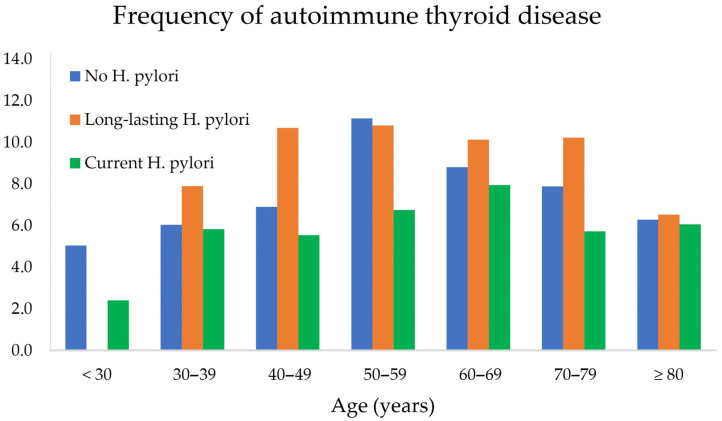
Frequency of autoimmune thyroid disease (AITD) according to age and *Helicobacter pylori* status.

Figure 1 illustrates the proportion of AITD as a function of age and *H. pylori* status. It can be noticed that AITD is clearly more frequent in patients with long-lasting *H. pylori* infection than in patients with current or no infection.

Results of the logistic regression analysis, after excluding the 306 patients with the past *H. pylori* infection successfully eradicated, are shown in Table 2. After adjusting for age, sex, BMI, and smoking status, the analysis revealed a positive association of long-lasting *H. pylori* infection with AITD (OR 1.34; 95%CI 1.13–1.60). The association was not significant for the current *H. pylori* infection (OR 0.99; 95%CI 0.64–1.57). Considering the overall *H. pylori* infection (without stratifying for gastritis subtypes) no significant risk difference was found.

When the analysis was conducted in patients with non-autoimmune TD, the association with *H. pylori* infection was neither observed in the case of long-lasting infection (OR 0.97; 95%CI 0.74–1.28) nor for the current infection (OR 0.86; 95%CI 0.66–1.15).

Patients with AITD were separated into those with Hashimoto’s thyroiditis and those with Graves’ disease. In both subgroups, the regression analysis showed a significant association with long-lasting *H. pylori* infection, but the effect size was greater in the latter (OR 1.94; 95%CI 1.20–3.15) than in the former (OR 1.32; 95%CI 1.06–1.64). On the contrary, the analysis did not highlight a significant association between *H. pylori* infection—either acute or chronic—with non-autoimmune TD.

Interestingly, after categorizing patients according to sex, the association between long-lasting *H. pylori* infection and AITD was detected as significant only among females (OR 1.39; 95%CI 1.12–1.72), while in males only a trend was observed, albeit a non-significant one (OR 1.19; 95%CI 0.63–2.23).

In an additional subanalysis with AITD patients stratified into those with a functionally compromised thyroid (maintained via levothyroxine therapy or methimazole) and those with euthyroid status, the association with long-lasting *H. pylori* infection remained in both groups (ORs 1.31 and 1.29, respectively).

## 4. Discussion

A number of studies has hinted that microorganisms play a significant etiological role in autoimmune disorders [19], including thyroid autoimmunity [38]. In the last decade, a growing literature has identified several bacterial taxa able to trigger Hashimoto’s thyroiditis and Graves’ disease [39]. Among microorganisms, *H. pylori* is also thought to increase the risk of developing AITD; however, there is a lack of definite consensus among endocrinologists about the robustness of this association and the possible interfering role of additional factors. The present study, conducted in a cohort of patients from Northern Sardinia, Italy, undergoing upper endoscopy for dyspeptic symptoms, tried to shed light for the first time on the role played by *H. pylori* infection according to gastritis phenotype, providing a number of interesting findings. First, a significant association was found between long-lasting *H. pylori* infection and AITD among female patients, independently of the gland’s functional status. The association was confirmed also adjusting for age, BMI, and smoking. More specifically, the association was observed with both Hashimoto’s and Graves’ diseases but not for non-autoimmune TD. Although the association was restricted to females, a trend was noted in males; however, we cannot exclude that the lower percentage of male patients in our cohort, and the consequent weak statistical power, could be the cause of this finding. Remarkably, only long-lasting *H. pylori* and not *H. pylori* infection as a whole showed an association with AITD. Current *H. pylori* infection did not show any significant association with TDs.

Overall, our results are partially in accord with data reported in the literature [20,25,27,30,31] supporting the claim that *H. pylori* infection is an independent risk factor for AITD. In particular, our results could help interpret the negative results obtained in other studies owing to the fact that the acute or chronic type of infection, based on histopathological features, was never taken into account.

The *H. pylori* and AITD association was generally corroborated by most case-control studies [24,29], although in other studies the association was confirmed only for patients infected by strains positive for the virulence CagA cytotoxic factor [27]. Instead, the prospective study by Arslan clearly showed that the association does not strictly depend on the presence of CagA-positive strains [31]. For instance, in 78 *H. pylori*-positive dyspeptic patients prospectively enrolled and 50 age-, gender-, and BMI-matched controls (*H. pylori*-negative dyspeptic patients), the association between CagA positivity and thyroid autoimmunity was evaluated. In that study, similarly to ours, the presence of *H. pylori* was established on gastric biopsies, and 55.1% of *H. pylori*-positive patients were CagA-positive (assessed by ELISA). The authors reported a significantly higher frequency of anti-TPO and Hashimoto’s thyroiditis in cases than in the control group, without any additional effect induced by the CagA status [31].

Even though in the present study the association with virulence factors could not be tested—owing to the unavailability of CagA typing—in a previous study, we reported that patients infected by *H. pylori* strains expressing CagA showed increased TPO Ab titers, despite the lack of a significant correlation between anti-*H. pylori* and anti-thyroid antibody titers. Vice versa, patients with hypothyroidism (thyroid-stimulating hormone (TSH) ≥ 3.5 μU/mL) had an increased frequency of anti-CagA Ab (*p* = 0.059) [40]. In a study by Bassi et al., the association between *H. pylori* infection and AITD was confined to Graves’ disease [41], whereas in our cohort, the association was found for both Graves’ disease and Hashimoto’s thyroiditis, although the effect size was greater in the former. Similarly, in the meta-analysis by Hou et al., the association of *H. pylori* infection was reported significant for both Graves’ disease (OR = 2.78, 95%CI: 1.68–4.61) and Hashimoto’s thyroiditis (OR = 2.16, 95%CI: 1.44–3.23) [32].

The association between *H. pylori* infection and AITD has also been described in Asian populations [30,32] and even in children [26], but whether the infection was current or long-lasting was not specified.

While most studies point to a positive association, two cross-sectional studies—based on *H. pylori* seropositivity—did not find any association [34,42]. 

The detection of serum IgG against *H. pylori* (or against *H. pylori* CagA-positive) provides a reliable assessment of current or prior *H. pylori* infection. Because the antibody tests remain positive long after successful treatment of the infection a positive test result cannot be used to ascertain whether the patient is infected or not. Lastly, the presence of antibodies against *H. pylori* can discern between a current or long-lasting infection [10]. Similarly, the urea breath test (UBT) and stool antigen test, although characterized by a very high accuracy in detecting the active infection, again cannot distinguish between a chronic–active infection from the presence of metaplasia/dysplasia/atrophy [10]. For example, in a previous study, the prevalence of hypothyroidism, or hyperthyroidism, was similar in *H. pylori*-positive and -negative subjects, where the infection was ascertained by UBT [43]. According to diagnostic methods used to detect *H. pylori* infection in the present study, we would have had a prevalence of AITD in *H. pylori*-positive patients and in *H. pylori*-negative patients of 49.6% vs. 50.4% (*p* > 0.05) by serology, and of 39.7% vs. 45.3% (*p* > 0.05) by the UBT/stool antigen test, respectively. The different tests used to detect *H. pylori* infection and the lack of adjustment for the gastritis phenotype may justify discrepancies among study findings. Our results indicate that the immune dysregulation determining the loss of specific tolerance for the thyroid, as a target organ, requires a long-lasting interaction between the microorganism and the host.

Mechanisms that could explain the association between *H. pylori* and AITD have been described by Cellini et al. [44]. The stomach and the thyroid gland are embryologically related [45], and a similarity between *H. pylori* and thyroid epitopes has been reported [42]. The damage of gastric cells by *H. pylori* infection exposes epitopes to the immune system, as demonstrated by the frequent association of anti-gastric parietal cell antibodies with AITD [46], thus potentially triggering an autoimmune response via molecular mimicry. However, while acute infection by *H. pylori* induces a strong proinflammatory immune process, the persistent infection induces instead a relatively weak type-2 immune reaction, driving the reprogramming of macrophage and dendritic-cell function [47], as well as an expansion of the T regulatory cell compartment [48]. In addition, T cells recognize *H. pylori* epitopes structurally similar to H/K/ATPase on the stomach parietal cells. This promotes the survival of *H. pylori* in the host but results in an increased probability of immunotolerance impairment.

However, even assuming a major role of cross-reactivity between anti-*H. pylori* antibodies and thyroid epitopes, the onset of an autoimmune response probably requires the presence of additional concomitant factors, including the genetic background of the host as well as the duration of the infection in the gastric epithelium. A study by Larizza et al. [26], which found a significant association between *H. pylori* infection and AITD, mostly in the presence of the HLA-DRB1*0301 allele—a well-known marker of autoimmunity—underlines the crucial role of a predisposing host genetic makeup in triggering and maintaining autoimmunity. The role of the HLA-DRB1*0301 allele may explain, at least in part, the negative results of a few studies that investigated the relationship between *H. pylori* infection and AITD.

This study has some limitations, including the retrospective design that was unable to assess a causal effect per se. In addition, in retrospective studies, the exposure to factors is not well controlled. However, the major exposure factor considered in this study for AITD occurrence, i.e., *H. pylori* infection, was ascertained by histology on at least four gastric specimens, and the presence of metaplasia/dysplasia/atrophy was used as a proxy for gastritis duration. Moreover, a number of patients undergoing upper endoscopy may have had a still-undiagnosed AITD, especially those without clinical evidence, although this kind of patient could have been missed in *H. pylori*-positive or -negative groups. Nonetheless, the large size of the cohort studied minimized the interference of these potential biases, making our findings reliable. An additional limitation is that the study was single-centered, and participants were characterized by a homogenous genetic background, hampering the generalizability of the results.

## 5. Conclusions

In conclusion, our results confirm an association between long-lasting *H. pylori* infection and AITD regardless of age, smoking status, and BMI in a population with a high prevalence of both conditions investigated for gastrointestinal symptoms. The association was restricted to female patients, although in males, an association trend was observed in aged participants.

## Figures and Tables

**Table 1:** Descriptive statistics of 8322 study participants according to the presence of autoimmune thyroid disease (AITD) and non-autoimmune thyroid disease (TD).

Variables	No TD (*n* = 7312)	AITD ^§^ (*n* = 562)	Non-AITD (*n* = 448)
Sex, *n* (%)			
Female	4437 (60.7)	511 (90.9)	391 (87.3)
Male	2875 (39.3)	51 (9.1) **	57 (12.7) **
Age, *n* (%)			
<30	956 (13.1)	36 (6.4)	14 (3.1) **
30–39	1089 (14.9)	65 (11.6)	44 (9.8) **
40–49	1226 (16.8)	86 (15.3)	81 (18.0)
50–59	1301 (17.8)	137 (24.4)	101 (22.5) **
60–69	1432 (19.6)	132 (23.5)	127 (28.3) **
70–79	1025 (14.0)	88 (15.7)	64 (14.3)
≥80	283 (3.9)	18 (3.2)	17 (4.0)
Body mass index, *n* (%)			
<25 kg/m^2^	4219 (57.7)	320 (56.9)	214 (47.8) **
25–29 kg/m^2^	2244 (30.7)	167 (29.7)	174 (38.8) **
≥30 kg/m^2^	849 (11.6)	75 (13.3)	60 (13.4)
Smoking status, *n* (%)			
Never smoker	3882 (53.1)	294 (52.3)	1094 (53.7)
Former smoker	314 (4.3)	25 (4.4)	111 (5.4) *
Current smoker	3116 (42.6)	243 (43.2)	834 (40.9)
*H. pylori* infection, *n* (%)			
No	3583 (53.2)	303 (51.8)	1977 (97.0)
Yes	3158 (46.8)	282 (48.2)	62 (3.0)
*H. pylori* status, *n* (%)			
No infection	4082 (55.8)	293 (52.1)	246 (54.9)
Long-lasting *H. pylori* infection	1519 (20.8)	149 (26.5) **	95 (21.2)
Current *H. pylori* infection	1456 (19.9)	93 (16.5)	83 (18.5)
Past *H. pylori* infection	255 (3.5)	27 (4.8)	24 (5.4)

* *p* < 0.05; ** *p* < 0.001; ^§^ TD = thyroid disease.

**Table 2:** Logistic regression analysis for autoimmune thyroid disease in 8016 study participants.

**Variables**	**AITD ^§^** **Unadjusted** **OR (95%CI)**	**AITD** **Adjusted** **OR 95%CI**	**Non AITD ^#^** **Adjusted** **OR 95%CI**
Sex			
Male	Ref.	Ref.	Ref.
Female	6.59 (4.94–8.81) **	6.81 (5.09–9.11) **	4.99 (3.60–6.92) **
Age			
<60	Ref.	Ref.	Ref.
≥60	1.17 (0.98–1.39)	1.18 (0.98–1.42)	1.40 (1.12–1.75)
Body mass index			
<25 kg/m^2^	Ref.	Ref.	Ref.
25–29 kg/m^2^	0.92 (0.76–1.12)	1.02 (0.84–1.25)	1.69 (1.33–2.15) *
≥30 kg/m^2^	1.13 (0.87–1.47)	1.18 (0.90–1.55)	1.61 (1.15–2.25) *
Smoking status			
Never smoker	Ref.	Ref.	Ref.
Former or current smoker	1.03 (0.87–1.22)	1.15 (0.97–1.38)	1.03 (0.82–1.29)
*H. pylori* infection			
No	Ref.	Ref.	Ref.
Yes	1.15 (0.96–1.36)	1.12 (0.94–1.34)	0.91 (0.73–1.14)
*H. pylori* status, *n* (%)			
No infection	Ref.	Ref.	Ref.
LL *H. pylori* infection ^§^	1.36 (1.11–1.67) **	1.34 (1.13–1.60) **	0.97 (0.74–1.28)
Current *H. pylori* infection	0.92 (0.72–1.17)	0.99 (0.64–1.57)	0.86 (0.66–1.15)

* *p* < 0.05; ** *p* < 0.01. ^§^ autoimmune thyroid disease; ^#^ non-autoimmune thyroid disease.

## Data Availability

Data will be available upon specific request to the corresponding author.
